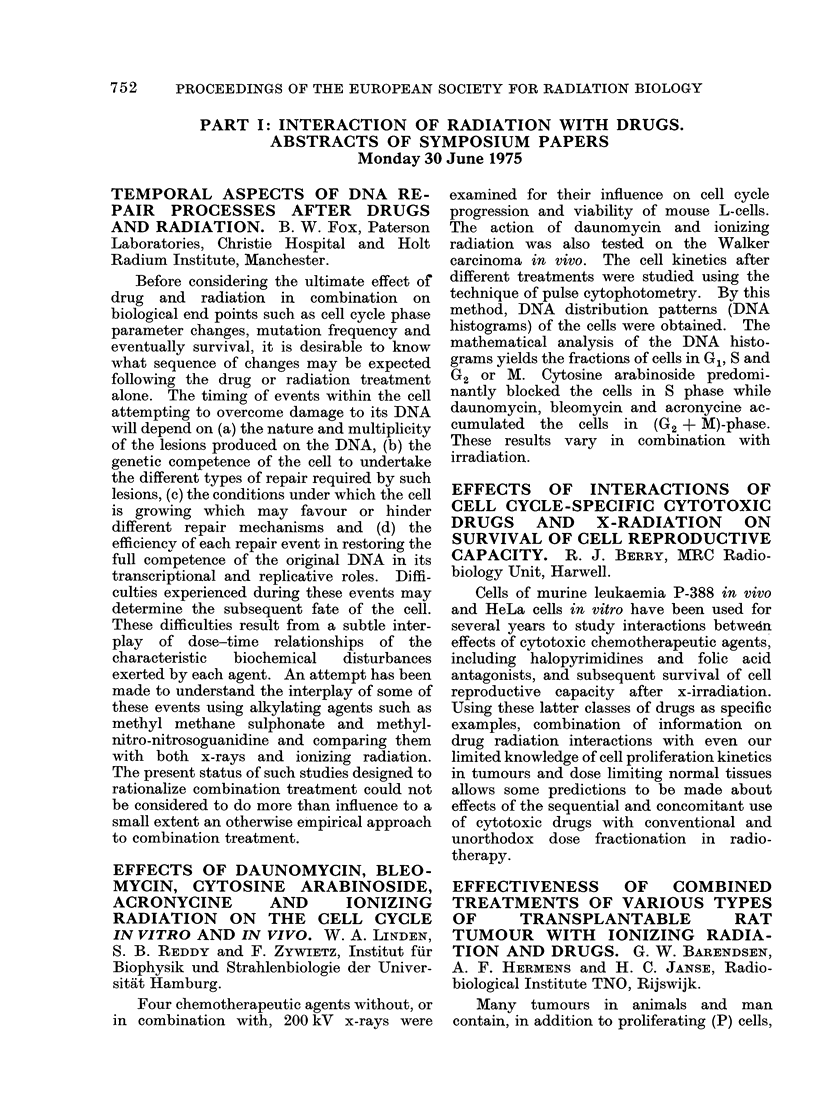# Proceedings: Temporal aspects of DNA repair processes after drugs and radiation.

**DOI:** 10.1038/bjc.1975.292

**Published:** 1975-12

**Authors:** B. W. Fox


### PART I: INTERACTION OF RADIATION WITH DRUGS. ABSTRACTS OF SYMPOSIUM PAPERS
### Monday 30 June 1975

**TEMPORAL ASPECTS OF DNA REPAIR PROCESSES AFTER DRUGS AND RADIATION.** B. W. Fox, Paterson Laboratories, Christie Hospital and Holt Radium Institute, Manchester.

Before considering the ultimate effect of drug and radiation in combination on biological end points such as cell cycle phase parameter changes, mutation frequency and eventually survival, it is desirable to know what sequence of changes may be expected following the drug or radiation treatment alone. The timing of events within the cell attempting to overcome damage to its DNA will depend on (a) the nature and multiplicity of the lesions produced on the DNA, (b) the genetic competence of the cell to undertake the different types of repair required by such lesions, (c) the conditions under which the cell is growing which may favour or hinder different repair mechanisms and (d) the efficiency of each repair event in restoring the full competence of the original DNA in its transcriptional and replicative roles. Difficulties experienced during these events may determine the subsequent fate of the cell. These difficulties result from a subtle interplay of dose–time relationships of the characteristic biochemical disturbances exerted by each agent. An attempt has been made to understand the interplay of some of these events using alkylating agents such as methyl methane sulphonate and methylnitro-nitrosoguanidine and comparing them with both x-rays and ionizing radiation. The present status of such studies designed to rationalize combination treatment could not be considered to do more than influence to a small extent an otherwise empirical approach to combination treatment.